# Current measures of metabolic heterogeneity within cervical cancer do not predict disease outcome

**DOI:** 10.1186/1748-717X-6-69

**Published:** 2011-06-09

**Authors:** Frank J Brooks, Perry W Grigsby

**Affiliations:** 1 Department of Radiation Oncology, Washington University School of Medicine, 4921 Parkview Place, Saint Louis MO 63110, USA; 2 Division of Nuclear Medicine, Mallinckrodt Institute of Radiology, Medical Center, Saint Louis MO, USA; 3 Department of Obstetrics and Gynecology, Washington University Medical Center, Saint Louis MO, USA; 4 Alvin J. Siteman Cancer Center, Washington University Medical Center, Saint Louis MO, USA

## Abstract

**Background:**

A previous study evaluated the intra-tumoral heterogeneity observed in the uptake of F-18 fluorodeoxyglucose (FDG) in pre-treatment positron emission tomography (PET) scans of cancers of the uterine cervix as an indicator of disease outcome. This was done via a novel statistic which ostensibly measured the spatial variations in intra-tumoral metabolic activity. In this work, we argue that statistic is intrinsically *non*-spatial, and that the apparent delineation between unsuccessfully- and successfully-treated patient groups via that statistic is spurious.

**Methods:**

We first offer a straightforward mathematical demonstration of our argument. Next, we recapitulate an assiduous re-analysis of the originally published data which was derived from FDG-PET imagery. Finally, we present the results of a principal component analysis of FDG-PET images similar to those previously analyzed.

**Results:**

We find that the previously published measure of intra-tumoral heterogeneity is intrinsically non-spatial, and actually is only a surrogate for tumor volume. We also find that an optimized linear combination of more canonical heterogeneity quantifiers does not predict disease outcome.

**Conclusions:**

Current measures of intra-tumoral metabolic activity are not predictive of disease outcome as has been claimed previously. The implications of this finding are: clinical categorization of patients based upon these statistics is invalid; more sophisticated, and perhaps innately-geometric, quantifications of metabolic activity are required for predicting disease outcome.

## Background

It is believed that cancerous tumors are intrinsically heterogeneous in many ways [1]. Experimentally quantified properties that exhibit significant variation within tumors include: gene expression [2], cell proliferation rate [3], degree of vascularization [4], and hypoxia [3,5]. When properties of tumors are assayed via an imaging technique such as positron emission tomography (PET), the question of quantifying biologically-functional heterogeneity becomes one of quantifying the spatial heterogeneity observed in grayscale images. In this case, one describes the arrangement of the various pixel intensities, with some arrangements subjectively appearing more heterogeneous than others. For example, the smooth gradation of a single bright spot to a darker background is intuitively less heterogeneous than the stark transitions seen by surrounding several clusters of the brightest pixels with only the darkest pixels. The goal of quantifying spatial heterogeneity is to objectively calculate a single statistic that indicates one pattern is a certain percentage more or less heterogeneous than another.

**Figure 1:**
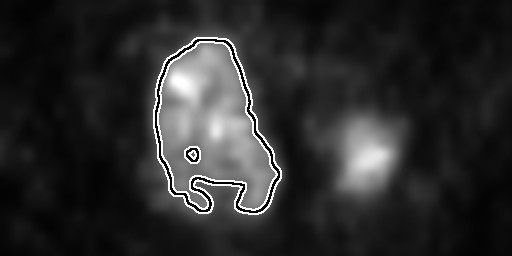
**Heterogeneity in an FDG-PET image**. A typical FDG-PET image of a cancer of the uterine cervix. The artificial boundary delineates the region of activity above the 40% of maximum intensity threshold. The heterogeneity within the tumor is evidenced by the very bright regions (higher metabolic activity) juxtaposed with relatively dark regions (lower metabolic activity). The undelineated bright spot to the right is a lymph node and is thus not included in the main tumor volume. The vertical edge of this image represents a length of 10 cm.

Although the applications of such a statistic to medical image processing and computational biology are broad, we focus our attention on the study of metabolic heterogeneity observed within cancers of the uterine cervix. In this case, cellular metabolism is assayed via the uptake of F-18 fluorodeoxyglucose (FDG), a glucose analog with a positron-emitting fluorine isotope [6]. Increased uptake of FDG implies increased metabolism of glucose [7], which is then indicated by an increased pixel intensity in the grayscale PET image. Upon inspection of a trans-axial, FDG-PET image of a typical cervical tumor (Figure 1), one can readily observe distinct regions of very bright pixel intensity near regions of lesser intensity, with each type of region being wholly contained within the bounds of the tumor. Since both the rate of proliferation [8] and the rate of healthy tissue invasion [7] are related to the rate of cellular metabolism, the motivation to quantify the observed variation in regional metabolism is obvious. One goal of such a study would be to investigate if this metabolic heterogeneity alone could serve as an predictor of disease outcome. Indeed, the major conclusion of precisely such a study is that intra-tumoral metabolic heterogeneity observed in pre-treatment cervical tumors predicts response to therapy and risk of recurrence [9].

In this work, we re-analyze the identical FDG-PET-derived data used in that previous study [9] and offer an alternative interpretation. Specifically, we argue that the novel measure employed in that work to quantify spatial heterogeneity of the grayscale PET images is intrinsically independent of spatial arrangement, and indeed is a surrogate for tumor volume. As such, it can offer no additional predictive capacity to that of tumor volume. Thus, the delineation of patients into distinct groups of post-treatment survival time via that heterogeneity measure is invalid. Additionally, we examine a similar data set and demonstrate that fundamental, non-spatial measures of heterogeneity applied to the FDG-PET assay of metabolic activity *do not *predict disease outcome. Finally, we discuss some implications of these results.

## Methods

### Analysis of Previously Published Data

In this work, we first re-analyze the same data originally analyzed in a previous heterogeneity-quantification study [9]. We briefly recapitulate the details of that prospective cohort study here. Patients underwent a pre-treatment, whole-body FDG-PET/CT scan. The pathologic diagnosis and histology were determined by pathologists at Washington University in St. Louis. All patients were treated with concurrent chemotherapy and radiation. A post-therapy FDG-PET/CT scan performed three months after completing the radiation treatment was used to evaluate the response to treatment. For our re-analysis of the 73 total patients, the 14 with persistent disease were combined with the 9 exhibiting new metastases into a single group of those having undergone unsuccessful treatment.

### Segmentation of Additional FDG-PET Imagery

The first task of analyzing imaged tumors is to delineate the tumors from the background (referred to as image segmentation). In the case of FDG-PET, the radiopharmaceutical is also taken up and metabolized by noncancerous cells, although to a lesser extent [10,11]. The typical result is an evidently stronger PET signal (tumor) surrounded by a weaker signal (non-tumor), with the possibility of additional *non*-tumorous bright-spots colocated with the bladder or rectum as undelivered radiopharmaceutical is cleared from the body [10]. As may be seen in Figure 1, the interface between the healthy and tumorous regions may not be stark, but rather nebulous as tumor cells invade healthy tissue in a diffuse fashion [12]. This is seen in the image as a smooth gradation from brighter pixels to dimmer ones. In order to objectively distinguish tumor from background, we employed the rule-of-thumb that, for a visually-selected, three-dimensional region of interest (ROI), any pixel brighter than 40% of the maximum ROI pixel brightness is to be considered part of the tumor. This 40% rule is based upon the observation that tumors defined as regions of greater than 40% of the maximum standard uptake value (SUV) of FDG both: colocate with those independently identified via visual analysis of computed tomography scans; and yield volumes consistent with published surgical series [13]. The SUV is a PET intensity measure that first has been converted to proper radiation units, then corrected for both radioactive decay and patient body mass [11]. For each patient, the net result is that every grayscale image pixel is multiplied by a single, positive constant. Because we seek to quantify *intra*-tumoral variation and since there is some debate as to the usefulness and validity of standard uptake values [14,15], we apply the 40% rule directly to the grayscale intensities.

A computer program to semi-automate the image segmentation process was written in Python v2.6.1 http://www.python.org/. As is ubiquitous in the field, the raw FDG-PET images are first processed through a white-balance-correcting, back-projection algorithm via the proprietary software native to the PET machine. The resulting DICOM image files are imported into our program via the pydicom library v0.9.3 http://code.google.com/p/pydicom/ and then converted to the 8-bit grayscale images via the Python Imaging Library v1.17 http://www.pythonware.com/products/pil/. No additional image preprocessing was implemented. Our program enables the user to rapidly target a region of the whole-body, trans-axial PET image set. Next, the program applies the 40% segmentation rule to all grayscale pixels in the targeted region (e.g., the pelvic region). A flood-fill algorithm is then applied to every pixel remaining in that region in order to determine the inter-pixel connectivity (or lack thereof). The result of this algorithm is a set of distinctly-bounded, contiguous objects. The user can then visually scan the objects and click to remove those few that are obviously (for sound anatomical reasons) not tumors. The typical end result is a 10 - 20 count stack of grayscale images representing trans-axial slices of a clearly-bounded tumor.

## Results

### Theory

**Figure 2:**
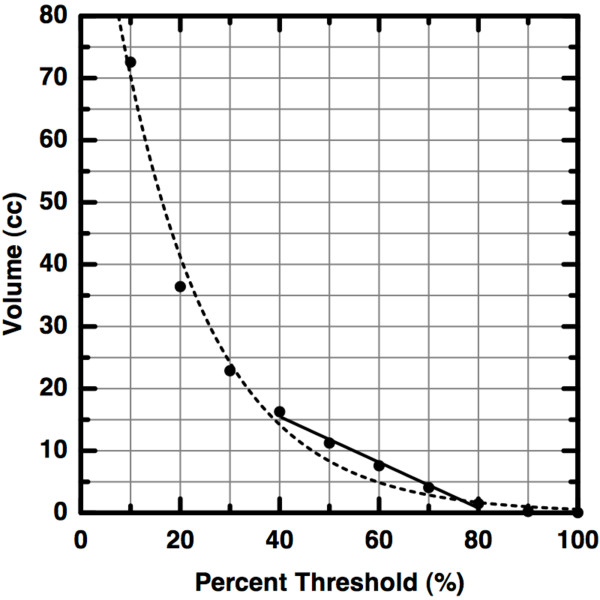
**Volume versus Threshold Curves**. A typical volume versus threshold curve (dots) from the data described in [9]. The tumor volume is defined to be those voxels with activity above 40% of the maximum activity. The slope of the line (-0.37 cc/%) of best fit between 40% and 80% was then used as a measure of intra-tumoral heterogeneity. This is the slope which we now argue does not predict disease outcome as was claimed in [9]. For reference, the best-fit exponential curve is also shown (dashed).

The original measure of heterogeneity presented in [9] was derived from a volume versus threshold curve for each tumor. In brief, a set of trans-axial image slices comprise a virtual tumor object in three-dimensional space. This object was segmented at increasingly high, grayscale intensity thresholds and the volume recorded at each threshold. The result of this process is a curve like the typical one shown in Figure 2. These curves were then linearized by first restricting the domain of the thresholding to be between 40 and 80 percent (inclusively) of the image maximum. The lower bound was chosen to guarantee that the tumor could be distinguished from the background (see Methods) and the upper bound was chosen to exclude the relatively small volumes represented by only the brightest pixels. The remaining coordinates were fit to a line and the resulting slope was used as a measure of heterogeneity. Greater magnitude of slope was interpreted to indicate greater heterogeneity, although we now argue that this is not the case.

**Figure 3:**
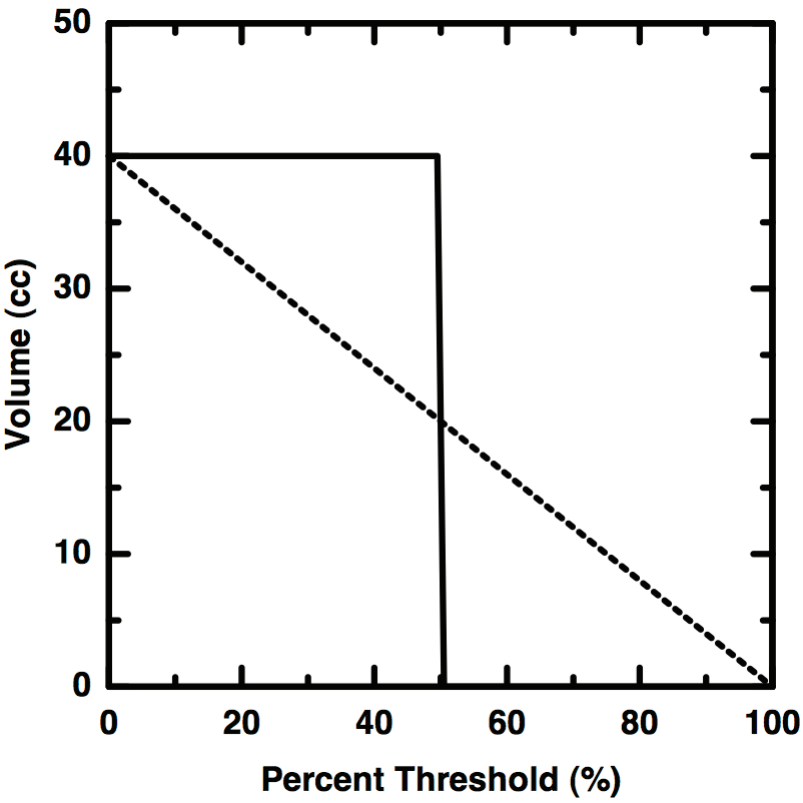
**Schematic Heterogeneity Curves**. The solid curve shows the nearly discontinuous drop (large slope) that must occur for a perfectly homogeneous volume of single activity level. The dashed line shows the curve expected for a volume containing equal numbers of each activity level possible. This heterogeneous scenario has a decreased slope. Thus, increasing slope implies increasing *homogeneity*. This is counter to the interpretation given in [9].

Consider a perfectly *homo*geneous volume consisting of only a single grayscale value. An example curve for such a scenario is shown as the solid curve in Figure 3. As the segmentation threshold is increased, no change is observed in the volume until the threshold becomes greater than the single value. Here, a virtually discontinuous drop to zero volume occurs. Next, consider a heterogeneous object, having the same volume as in the previous example, but with each of *N *> 1 grayscale values represented in equal number. In this case, the same change in volume is spread over a greater threshold change. We therefore observe that as more grayscale values are used, heterogeneity increases and slope *decreases*. Because each grayscale value is represented equally, the change in volume for a given change in percent threshold is constant (Figure 3 (dashed)). Therefore, a perfectly linear volume versus threshold curve implies maximal heterogeneity over multiple grayscale values.

It is important to point out that in the scheme described above, the numeric value of the slope is independent of spatial arrangement. For example, the set of grayscale values representing the tumor could be rearranged such that each value resides at a new 3D Cartesian coordinate. In other words, it is possible to "draw" various artificial objects by purposefully placing selected grayscale values at desired coordinates. However, the *number *of each distinct grayscale value remains constant, regardless of where in the object those values may reside. Since the volume of the tumor object ultimately was calculated by counting pixels above a given threshold, that volume does not change even when the tumor object is destroyed via rearrangement. Thus, any measure of heterogeneity given by the slope is only of the diversity of intensity values, not in spatial arrangement of those values.

### Critique of Previously Published Results

In a stack of trans-axial, FDG-PET images, a region of interest fully containing the tumor is first selected by a trained clinician. This is the region of interest that is successively thresholded and the volume of the region remaining after thresholding is computed. Let *V_A_*(*T*) = *V*_*A*0 _*e*^-*λT *^approximate a typical, observed volume (*V*) versus percent threshold (*T*) curve for patient *A *(see Figure 2). At zero percent threshold, *V_A_*(0) = *V*_*A*0_, the total volume of the initial target region. It is straightforward to show that the slope of the line between a minimum, tumor-defining threshold *T_m _*and twice that threshold (e.g, 40% and 80%) is *s_A _*= (*V_A_*(*T_m_*)/*T_m_*) · (*V_A_*(*T_m_*)/*V*_*A*0 _- 1). We now wish to compare this slope (ostensibly a measure of heterogeneity) to that of a second patient, *B*, where *V*_*B *_(*T*) = *V*_*B*0 _*e*^-*μT*^. From the 73 available *V *(*T*) curves, we observed that, save for extremely large tumor volumes (greater than 150 cm^3^), the total volume of tumor exhibiting pixel intensities greater than 80% of the maximum observed intensity is typically very small (≈3 cm^3^). Thus, the end points of the linearization are approximately equal *for every patient*. Therefore, , from which it is seen that . Proceeding as before, and employing this approximation, one may show that the change in slope is Δ*s *≡ |*s_A _*- *s_B_*| = |*V_B_*(*T_m_*) - *V_A_*(*T_m_*)|/*T_m _*≡ Δ*V *(*T_m_*)/*T_m_*. In words, the previously published measure of intra-tumoral heterogeneity is directly proportional to the pre-treatment tumor volume. It is important to note that this result depends only upon the *measured *40% tumor volumes, and in no way depends upon the decay rate or closeness of fit of either exponential curve.

**Figure 4:**
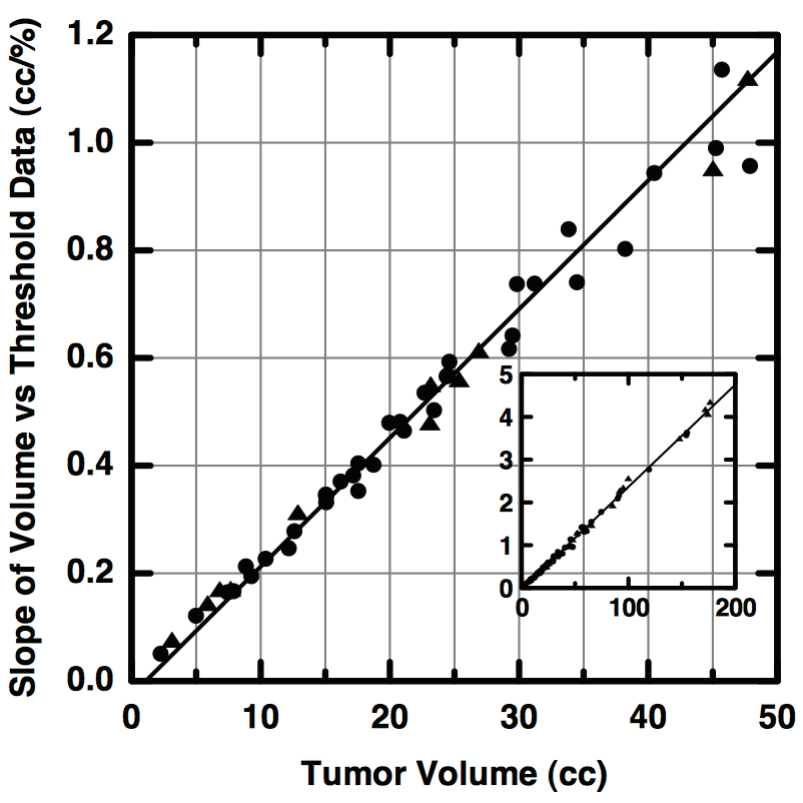
**A Volume Surrogate**. A previously published measure of intra-tumoral heterogeneity is plotted versus tumor volume for patients who underwent successful (circles) or unsuccessful (triangles) therapy. Observe that the heterogeneity measure is directly proportional to volume and there is a lack of clustering of patients into distinct groups with differing disease outcome. As seen in the inset, the trend persists over three orders of magnitude. The inset axes have the same units as in the primary plot.

The linear proportionality derived above is seen in the original FDG-PET data. As described in [9], we plotted the total volume (in cm^3^) of the target region with pixel intensities greater than a given percent threshold versus percent threshold. We then computed the least-squares linear regression for points between 40% and 80% thresholds. The magnitude of the slope is plotted versus the tumor volume (i.e., that defined at 40% threshold) in Figure 4. As predicted, it is clearly seen that the slope magnitude is linearly proportional to tumor volume. Therefore, the previously published delineation between unsuccessfully- and successfully-treated patient groups is based exclusively upon tumor volume, not upon any additional measure of heterogeneity. Larger volumes intuitively imply long-duration, aggressive tumor progress. Thus, the simplest explanation of a statistically-significant, predictive result (in [9]) is that the relatively small number of patients with new or persistent cancer tended to have larger pre-treatment tumor volumes. In other words, the apparent statistical significance is no more than the expected artifact arising from the inappropriate use of the standardized permutation test (p-test) upon groups with greatly differing numbers of members.

**Figure 5:**
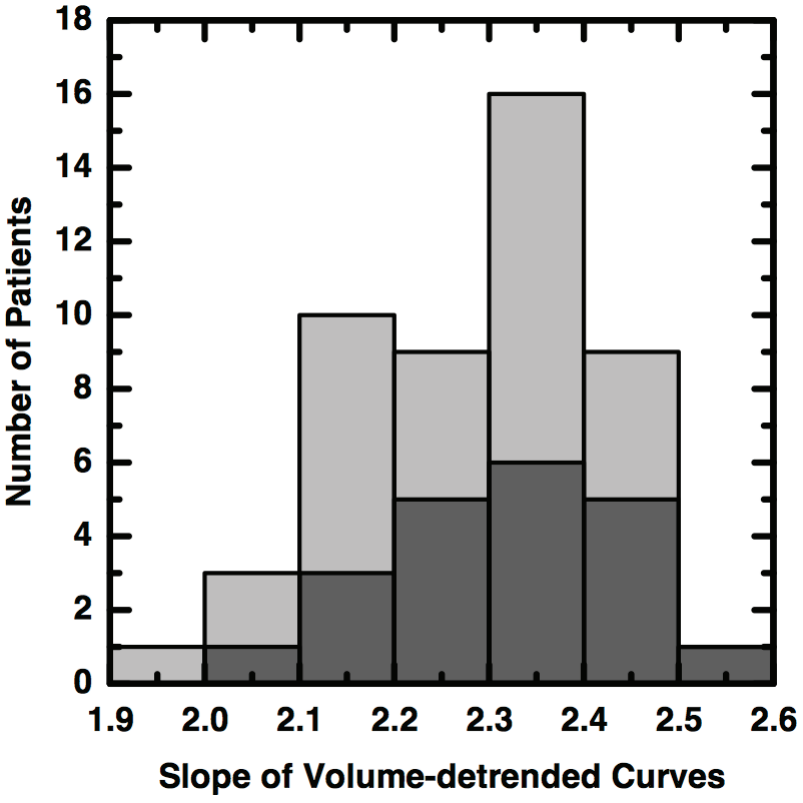
**No Predictive Value**. Histograms of the volume-detrended slopes for patients who underwent successful (light shading) or unsuccessful (dark shading) therapy. The overlapping histograms indicate that the ostensible measure of distinguishing intra-tumoral heterogeneities actually has the same mean value for every patient, differing only by random noise, and thus does not predict disease outcome.

An important consequence of the finding that Δ*s *∝ Δ*V *is that the slopes computed for similar volumes should themselves be similar, differing only by random noise. To see this, we first detrended the slopes by dividing each by the 40% tumor volume. This is identical to having first plotted the *percent volume *versus percent threshold and computing the slope of the best-fit line. The dimensionless, volume-detrended slopes were pooled and then a histogram bin width of 0.1 was computed via a commonly-used, optimal bin-width formula [16]. The slopes were separated into distinct groups based upon *a priori *knowledge of patient outcome. A histogram of volume-detrended slopes was created for each group and is shown in Figure 5. There, it is clearly seen that the group which underwent successful treatment (light shading) almost completely overlaps that which underwent unsuccessful treatment (dark shading). Each group differs from a single mean of 2.3 by the same standard deviation, 0.13. This important observation, that the volume-detrended slopes are essentially identical for every patient, implies that the previously published measure of intra-tumoral heterogeneity is not in any way predictive of disease outcome.

In an effort to verify this result, we studied the FDG-PET imagery of 47 recently-examined patients that did not appear in the previously published study. The images were again obtained as described in [9] but segmented as described in the Methods section. We computed the volume-detrended slopes as before.

Again, we found no distinguishing capacity whatsoever between the successfully treated patients, where the mean slope is 2.20, and the unsuccessfully treated patients where the mean slope is 2.23.

### Extended Heterogeneity Analysis

Previous arguments imply that the volume versus threshold slope is sensitive to the distribution of grayscale intensities of the trans-axial image stack. We therefore chose to investigate the relation between these distributions and disease outcome via the fundamental quantifiers of distributions: the standard deviation, skewness and kurtosis. Each of these quantifiers describes a unique quality of non-spatial heterogeneity. The standard deviation indicates the number of unique grayscale values comprising the image stack; that is, the number of different levels of metabolic activity observed. The kurtosis indicates the relative strength of those metabolic levels since a distribution with only a single, sharp peak (higher kurtosis) indicates a favored metabolic activity level. The skewness indicates the pervasiveness of activity levels. For example, an overall brighter distribution (negatively skewed) implies that the majority of tumor volume exhibits relatively higher metabolic activity whereas a skewness of zero indicates equal volumes of activities above and below the mean activity.

**Figure 6:**
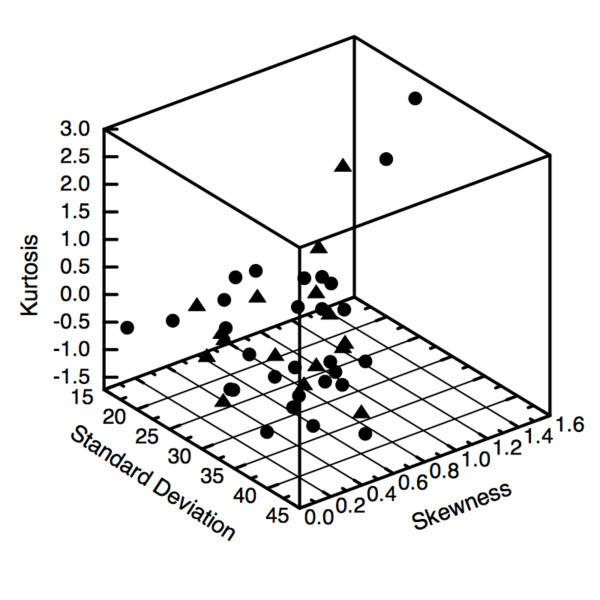
**Quantifier phasespace**. A phase space of intuitive, non-spatial quantifiers of heterogeneity is shown. Each point has a standard deviation, skewness and kurtosis coordinate. As is evident in the plot, and confirmed via principal component analysis, there is no delineation between patients who underwent successful (circles) or unsuccessful (triangles) radiotherapy.

Since each of the fundamental quantifiers describing the distribution of FDG-PET intensities represents an independent, *biological *aspect of the tumor, it seems reasonable to us that they are members of a basis set of heterogeneity-describing statistics. In other words, we suggest that any feasible non-spatial indicator of heterogeneity would have to in some way depend upon the standard deviation, skewness and kurtosis. We computed these quantifiers for the 8-bit grayscale intensity distributions for each of the 47 recently-examined patients. We then constructed a three-dimensional phase space where each patient is represented by a point having a standard deviation, skewness and kurtosis coordinate. Each point in that space is then given a unique symbol corresponding to patient outcome after chemoradiotherapy with curative intent. In Figure 6, it is seen that the patients free of cancer after therapy (circles) are well-mixed with those for whom therapy was unsuccessful (triangles), and no obvious clustering of the patient groups is apparent. To explore whether any predictive information can be obtained from the non-spatial metabolic activity quantifiers, we performed a principal component analysis. The standard deviation, skewness, and kurtosis for each of 47 patients comprise the rows of the 3 × 47 matrix of observations. As is described in many textbooks [17], we then compute the unit-magnitude eigenvectors of the mean-detrended covariance matrix to obtain the single variable representing the maximal use of information within the initial variables. We found that a new variable, *ψ *= 0.999 standard deviation - 0.010 skewness - 0.033 · kurtosis, best described the variation in phase space. Since the disease outcomes are known, we computed the value of *ψ *for each patient and performed a standardized permutation test of significance (p-test). The mean values of *ψ *for patients undergoing successful or unsuccessful treatment are 30.4 (p = 0.36) and 28.8 (p = 0.24), respectively. The two-sided p-values given here indicate that our default assumption that the mean of one group equals the mean of the other cannot be rejected. In other words, these relatively large p-values are consistent with our earlier observation (seen in Figure 6) that there is no substantial difference between the values of *ψ *for each treatment group. Thus, our conclusion is that the optimal linear combination of the non-spatial metabolic quantifiers does not predict disease outcome any better than random chance.

From the corresponding eigenvalues, we compute that ≈98% of the total variation in phase space is represented by the standard deviation alone. This high percentage indicates that more sophisticated, non-spatial measures of heterogeneity--which we assert ultimately are based upon the fundamental quantifiers--are unlikely to improve upon the standard measure of uncertainty. In other words, the standard deviation alone is a reasonable non-spatial measure of the variation in metabolic activity. Thus, we suggest that the textbook usage of the standard deviation as the uncertainty in the mean value is adequate when computing statistics, such as the total glycolytic volume, which are spatially averaged over the entire tumor volume.

A potential concern lies in our definition of patient groups, where the unsuccessfully treated group is the union of those patients having post-treatment persistent cancer with those having post-treatment new metastases. In an effort to avoid any bias due to pre-existing metastases, we performed both the re-analysis of existing data as well as our entire principal component analysis again. We first eliminated those with new metastases from the unsuccessfully treated group. We then computed the volume-detrended slopes described earlier and again found that mean value for the successfully treated group (2.28) is nearly identical to that (2.32) of the unsuccessfully treated group. Thus, bias due to inclusion of patients with new metastases does not explain the lack of predictive capacity of the previously published measure of heterogeneity. We now explore the potential effect of this bias in our principal component analysis. Proceeding as before, we compute a new *ψ *variable for the truncated matrix of observations, excluding patients with new metastases. The mean values of *ψ *for patients undergoing successful or unsuccessful treatment are then 30.4 (p = 0.51) and 31.7 (p = 0.38), respectively. We again see no substantive difference between the mean values for each group and thus conclude that patients with new metastases did not bias our previous result that non-spatial metabolic quantifiers do not predict disease outcome.

## Discussion

It is important that we immediately point out that we are not claiming that intra-tumoral metabolic heterogeneity does not exist. Indeed, we presume that metabolic activity can vary significantly throughout a tumor. In a younger, pre-vascularized tumor, such variations are likely due to a non-constant, diffusion-limited nutrient density [18]. In a mature tumor, these variations could be due to necrosis [18] or even steric constraints imposed by the spatially-randomized, densely-packed nature of newly-formed vascularization networks [19]. In order to measure a genuine heterogeneity in a stack of images, one must be able to distinguish a single volume element (voxel) from another. The minimum detectable inter-voxel difference is determined by the noise intrinsic to the FDG-PET assay. The noise in a typical 3D FDG-PET image reconstructed via filtered back-projection has been estimated to be 1.5 kBq/mL [20]. This is only 3% of the ≈50 kBq/mL mean activity of all tumor voxels defined above 40% intensity threshold in our extended heterogeneity study. This implies that the FDG-PET assay can distinguish relatively small changes in the metabolism of tumor cells averaged over a typical PET image voxel. We therefore conclude that the non-predictive nature of bulk heterogeneity statistics is not due to either a genuine lack of variation in metabolic activity or the poor resolution of this variation.

Instead, our results imply that that quantification of tumor composition via FDG-PET remains a challenging, open problem to be solved. We maintain that a shift of focus from tumor composition to shape and location offers immediate potential for improved clinical therapy. Consider that the uncertainty in the anatomical placement of brachytherapy radiation sources via a standard gynecological implant is at least several millimeters. This is the same order of spatial uncertainty in FDG-PET-assayed tumors where the side length of a cubical voxel is typically ≈4 mm. Also, as the computation of radiation fields is rapidly becoming more accurate and more computationally-accessible [21], it is feasible that more precise, *geometric *quantification of metabolic variations will directly yield more effective treatment plans. For example, it could be the case that tumors of a particular shape or asymmetry are indicative of disease outcome [22,23]. These geometric qualities can be quantified readily via the well-known techniques common to image texture analysis [24] or the physics of particle systems [25].

## Conclusions

We have shown that neither the currently accepted measure, nor other reasonable non-spatial measures, of intra-tumoral metabolic heterogeneity within cervical cancer are predictive of disease outcome. This is directly counter to a previously published claim. We have given a brief mathematical explanation of why that claim is erroneous and have supported our argument with the results of both a re-analysis of the originally published data and a fundamental statistical analysis of a similar data set. Our findings have immediate impact upon clinical research and treatment. The use of currently-accepted, non-spatial quantifiers of intra-tumoral metabolic heterogeneity as a means to categorize patients into groups predicted to be successfully or unsuccessfully treated is invalid. Thus, more sophisticated, and perhaps innately-geometric, quantifications of metabolic activity are required for predicting disease outcome.

## Competing interests

Frank J. Brooks has no conflicts of interests. Perry W. Grigsby has no conflicts of interests.

## Authors' contributions

FJB conceived and drafted the manuscript as well as performed all mathematical analyses. PWG designed the protocol for the interpretation the FDG-PET images, acquired the volumetric data presented, and provided crucial medical and anatomical insight into the analyzed data and imagery. Both FJB and PWG read and approved the final manuscript.

## References

[B1] Heppner GH Tumor heterogeneity Cancer Res 1984 44 6 2259 65 6372991

[B2] Zhao S Kuge Y Mochizuki T Takahashi T Nakada K Sato M Takei T Tamaki N Biologic correlates of intratumoral heterogeneity in 18F-FDG distribution with regional expression of glucose transporters and hexokinase-II in experimental tumor J Nucl Med 2005 46 4 675 82 15809491

[B3] Pugachev A Ruan S Carlin S Larson SM Campa J Ling CC Humm JL Dependence of FDG uptake on tumor microenvironment Int J Radiat Oncol Biol Phys 2005 62 2 545 53 10.1016/j.ijrobp.2005.02.009 15890599

[B4] Révész L Siracka E Siracky J Delides G Pavlaki K Variation of vascular density within and between tumors of the uterine cervix and its predictive value for radiotherapy Int J Radiat Oncol Biol Phys 1989 16 5 1161 3 10.1016/0360-3016(89)90274-5 2715063

[B5] Picchio M Beck R Haubner R Seidl S Machulla HJ Johnson TD Wester HJ Reischl G Schwaiger M Piert M Intratumoral spatial distribution of hypoxia and angiogenesis assessed by 18F-FAZA and 125I-Gluco-RGD autoradiography J Nucl Med 2008 49 4 597 605 10.2967/jnumed.107.046870 18344437

[B6] Bailey DL Positron emission tomography: basic sciences 2005 New York: Springer

[B7] Gatenby RA Gillies RJ Why do cancers have high aerobic glycolysis? Nat Rev Cancer 2004 4 11 891 9 10.1038/nrc1478 15516961

[B8] Vander Heiden MG Cantley LC Thompson CB Understanding the Warburg effect: the metabolic requirements of cell proliferation Science 2009 324 5930 1029 33 10.1126/science.1160809 19460998 PMC2849637

[B9] Kidd EA Grigsby PW Intratumoral metabolic heterogeneity of cervical cancer Clin Cancer Res 2008 14 16 5236 41 10.1158/1078-0432.CCR-07-5252 18698042

[B10] Cook GJR Bailey ea Dale L Artefacts and Normal Variants in Whole-Body PET and PET/CT Imaging Positron emission tomography: basic sciences 2005 1 Springer 281 293

[B11] Wahl RL Wahl RL Standardized Uptake Values Principles and Practice of PET and PET/CT 2009 2 Wolters Kluwer Health

[B12] Weinberg RA The biology of cancer 2007 New York: Garland Science

[B13] Miller TR Grigsby PW Measurement of tumor volume by PET to evaluate prognosis in patients with advanced cervical cancer treated by radiation therapy Int J Radiat Oncol Biol Phys 2002 53 2 353 9 10.1016/S0360-3016(02)02705-0 12023139

[B14] Keyes JW Jr SUV: standard uptake or silly useless value? J Nucl Med 1995 36 10 1836 9 7562051

[B15] Paulino A Johnstone P Does SUV stand for silly useless value? Int J Radiat Oncol Biol Phys 2004 60 3 1006

[B16] Izenman AJ Recent developments in nonparametric density-estimation Journal of the American Statistical Association 1991 86 413 205 224 10.2307/2289732

[B17] Lay DC Linear algebra and it's applications 2006 3 Boston: Pearson/Addison-Wesley21660123

[B18] Gerlee P Anderson ARA Evolution of cell motility in an individual-based model of tumour growth J Theor Biol 2009 259 67 83 10.1016/j.jtbi.2009.03.005 19285513 PMC2706369

[B19] Jain RK Molecular regulation of vessel maturation Nat Med 2003 9 6 685 93 10.1038/nm0603-685 12778167

[B20] Schmidtlein CR Beattie BJ Bailey DL Akhurst TJ Wang W Gönen M Kirov AS Humm JL Using an external gating signal to estimate noise in PET with an emphasis on tracer avid tumors Phys Med Biol 2010 55 20 6299 326 10.1088/0031-9155/55/20/016 20924132

[B21] Thomadsen BR Williamson JF Rivard MJ Meigooni AS Anniversary paper: past and current issues, and trends in brachytherapy physics Med Phys 2008 35 10 4708 23 10.1118/1.2981826 18975716

[B22] O'Sullivan F Roy S O'Sullivan J Vernon C Eary J Incorporation of tumor shape into an assessment of spatial heterogeneity for human sarcomas imaged with FDG-PET Biostatistics 2005 6 2 293 301 10.1093/biostatistics/kxi010 15772107

[B23] Mayr NA Yuh WTC Taoka T Wang JZ Wu DH Montebello JF Meeks SL Paulino AC Magnotta VA Adli M Sorosky JI Knopp MV Buatti JM Serial therapy-induced changes in tumor shape in cervical cancer and their impact on assessing tumor volume and treatment response AJR Am J Roentgenol 2006 187 65 72 10.2214/AJR.05.0039 16794157

[B24] Jähne B Digital image processing 2005 6th rev. and ext Berlin: Springer

[B25] Arfken GB Weber HJ Mathematical methods for physicists 2005 6 Boston: Elsevier

